# Co-Circulation of Dengue Virus Serotypes 1, 2, and 3 during the 2022 Dengue Outbreak in Nepal: A Cross-Sectional Study

**DOI:** 10.3390/v15020507

**Published:** 2023-02-11

**Authors:** Sandesh Rimal, Sabin Shrestha, Kishor Pandey, Thanh Vu Nguyen, Parmananda Bhandari, Yogendra Shah, Dhiraj Acharya, Nabaraj Adhikari, Komal Raj Rijal, Prakash Ghimire, Yuki Takamatsu, Basu Dev Pandey, Stefan Fernandez, Kouichi Morita, Mya Myat Ngwe Tun, Shyam Prakash Dumre

**Affiliations:** 1 Central Department of Microbiology, Tribhuvan University, Kathmandu 44601, Nepal; 2 Central Department of Zoology, Tribhuvan University, Kathmandu 44601, Nepal; 3 Institute of Tropical Medicine, DEJIMA Infectious Disease Research Allience, Nagasaki University, Nagasaki 852-8523, Japan; 4 Sukraraj Tropical and Infectious Diseases Hospital, Teku, Kathmandu 44600, Nepal; 5 Seti Provincial Hospital, Kailali 10900, Nepal; 6 Cleveland Clinic, Florida Research and Innovation Center, Port Saint Lucie, FL 34987, USA; 7 Department of Molecular Epidemiology, Institute of Tropical Medicine, Nagasaki University, Nagasaki 852-8523, Japan; 8 Armed Forces Research Institute of Medical Sciences (AFRIMS), Bangkok 10400, Thailand

**Keywords:** dengue virus, dengue serotypes, dengue outbreak 2022, multiple serotypes, Nepal

## Abstract

The largest dengue outbreak in the history of Nepal occurred in 2022, with a significant number of casualties. It affected all 77 districts, with the nation’s capital, Kathmandu (altitude 1300 m), being the hardest hit. However, the molecular epidemiology of this outbreak, including the dengue virus (DENV) serotype(s) responsible for this epidemic, remain unknown. Here, we report the epidemic trends, clinico-laboratory features, and virus serotypes and their viral load profiles that are associated with this outbreak in Nepal. Dengue-suspected febrile patients were investigated by routine laboratory, serological, and molecular tools, including a real-time quantitative polymerase chain reaction (qRT-PCR). Of the 538 dengue-suspected patients enrolled, 401 (74.5%) were diagnosed with dengue. Among these dengue cases, 129 (32.2%) patients who required hospital admission had significant associations with myalgia, rash, diarrhea, retro-orbital pain, bleeding, and abdominal pain. DENV-1, -2, and -3 were identified during the 2022 epidemic, with a predominance of DENV-1 (57.1%) and DENV-3 (32.1%), exhibiting a new serotype addition. We found that multiple serotypes circulated in 2022, with a higher frequency of hospitalizations, more severe dengue, and more deaths than in the past. Therefore, precise mapping of dengue and other related infections through integrated disease surveillance, evaluation of the dynamics of population-level immunity and virus evolution should be the urgent plans of action for evidence-based policy-making for dengue control and prevention in the country.

## 1. Introduction

Dengue is a mosquito-borne viral infection caused by any one of the four dengue virus serotypes (DENV-1 through -4) and a significant global health threat that affects 2.4 billion people worldwide [1]. A global estimate based on 2010 reported more than 100 million cases of symptomatic dengue from >128 countries across the globe [2]. Most dengue patients are either asymptomatic or present with mild, flu-like symptoms, while a small proportion of cases may progress into a life-threatening form of the disease known as severe dengue [3,4]. Dengue has a moderate-to-low mortality rate; however, the mortality rate among severe dengue patients may rise significantly with inadequate clinical management [3]. Despite growing research on potential severity predictors, no definitive predictors of severe dengue resulting in unwanted hospitalization and fatalities have been identified [5,6,7].

The global epidemiological trend of dengue shows an escalation, with an expansion to previously non-endemic regions/countries [1,2,8]. Globally, the number of dengue cases increased eight times over the last two decades (0.5 million in 2000 to 4.2 million in 2019) [9]. The South-East Asia region carries more than half of the global dengue burden, with temporal extensions to new member states such as East Timor, Bhutan, and Nepal in the past two decades [3,10]. 

Nepal, a Himalayan country with diverse ecological, topographical, and climatic conditions, is a unique geographical setting in which to understand dengue epidemiology. After its first appearance in 2004 [11], Nepal experienced a series of dengue outbreaks that ranged from small-scale localized transmissions to larger/nation-wide epidemics [12,13,14,15,16,17,18,19]. Major large-scale outbreaks occurred in 2010, 2013, 2016, 2017, and 2019, indicating a loose cyclical trend [16,20]. After the historical 2019 outbreak with 17,992 cases, dengue reporting was substantially overshadowed by the COVID-19 pandemic [13,17,20,21]. This period served as a prelude to an unprecedented disaster, and in 2022, the largest dengue epidemic in the history of Nepal occurred, with a rapid surge in cases in the weeks of the 8th to the 26th of August, although sporadic cases were observed as early as January [20,22,23]. Responding to this crisis, the Epidemiology and Disease Control Division (EDCD) conducted ‘search and destroy’ campaigns and public awareness activities to control dengue. In 2022, there were 54,784 cases and 88 casualties due to dengue across all 77 districts of Nepal according to EDCD [22]. 

In the past 10–12 years, demographical profiles suggest that dengue has migrated substantially to higher altitudes in Nepal [14,15,16]; however, due to the lack of virus surveillance in mosquitoes and adequate molecular profiling of circulating DENV serotypes, it is difficult to precisely estimate the altitudes to which the virus has migrated. This has greatly challenged efforts to control dengue, and recent outstanding issues such as climate change are thought to play roles in transmission dynamics, disease magnitude, and geographical range. DENV serotype(s) displacement might have played a crucial role in Nepal’s unusual epidemic patterns, including in the 2022 epidemic [20,22,24]. Despite the large number of cases and casualties resulting from the 2022 dengue epidemic in Nepal, no molecular information had been available on the DENV serotype(s) causing this epidemic, although government authorities published periodic situation reports containing the overall epidemiological information. Here, we report the clinical and laboratory features, spatiotemporal characteristics, and serotype distribution of the 2022 dengue epidemic Nepal.

## 2. Materials and Methods

### 2.1. Country Context and the Outbreak

**Figure 1:**
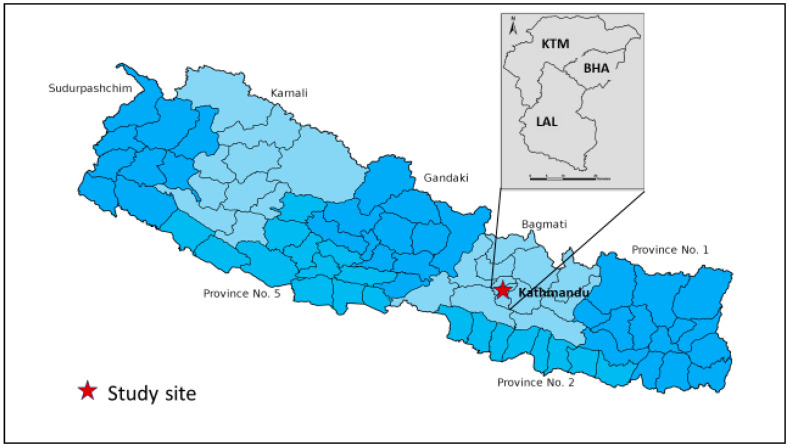
Map of Nepal showing the study site.

Nepal is a landlocked country bordering India on three sides (south, east, and west) and China on the north. The total population of Nepal according to the 2021 census is 29 million, with a population growth rate of 0.93%. which inreased by 10.2% over the past 10 years. Nepal is divided politically into seven provinces, with 77 districts, and 753 local governments (Figure 1). Topographically, Nepal is divided into three regions: Terai, Hills, and Mountains. Over half of the population lives in the India-bordering Terai region of Nepal, where cross-border migration is high and vector-borne diseases are endemic [12,16,25]. Although dengue is not a new disease in Nepal anymore, its changing epidemiological pattern, with frequent serotype switching, migration to higher altitudes, and nationwide expansion are certainly matters of serious concern. Another flavivirus, (Japanese encephalitis virus [JEV]), underwent a similar expansion toward higher altitudes before immunization started in Nepal. Dengue case numbers during the 2022 outbreak rapidly increased starting in July, coinciding with the rainy season, and meteorologists forecasted above-normal monsoon rains that year (the monsoon started earlier and remained for longer than previous years) [26]. Furthermore, the highest number of dengue cases was reported from Kathmandu, located at an altitude of 1300 m above sea level, which is also quite unusual in a global context. The capital city, Kathmandu, has an urban population inhabiting three districts (Kathmandu, Bhaktapur, and Lalitpur). The population of Kathmandu comprises three million inhabitants and a large number of people who migrate from across the country for work, education, or settlement, generating ample opportunities for infectious disease transmission. Therefore, there is a great need of in-depth understanding of the dengue epidemic at this altitude.

### 2.2. Study Design and Setting

This hospital-based cross-sectional study was conducted at Sukraraj Tropical and Infectious Disease Hospital (STIDH), located in the capital city of Nepal (Figure 1). STIDH is a public referral hospital for the diagnosis and treatment of tropical and infectious diseases in Nepal, which dengue-suspected patients from across the country visit for diagnosis and clinical care. Kathmandu was the hardest-hit area during the 2022 dengue epidemic, and STIDH had the highest dengue patient flow.

### 2.3. Patient Enrollment, Questionnaire, and Laboratory Analysis

Dengue-suspected patients of any age and sex were enrolled after obtaining written informed consent from each patient or their guardian in the case of children (Figure 1). A standardized questionnaire was administered to collect the baseline demographic data: age, sex, address, history of past infection, and travel history. A 3–5 mL blood sample was collected and subjected to serum separation for chemistry, serology and further molecular analysis, while ethylenediamine tetraacetic acid (EDTA) blood was prepared for hematological analysis. 

**Figure 2:**
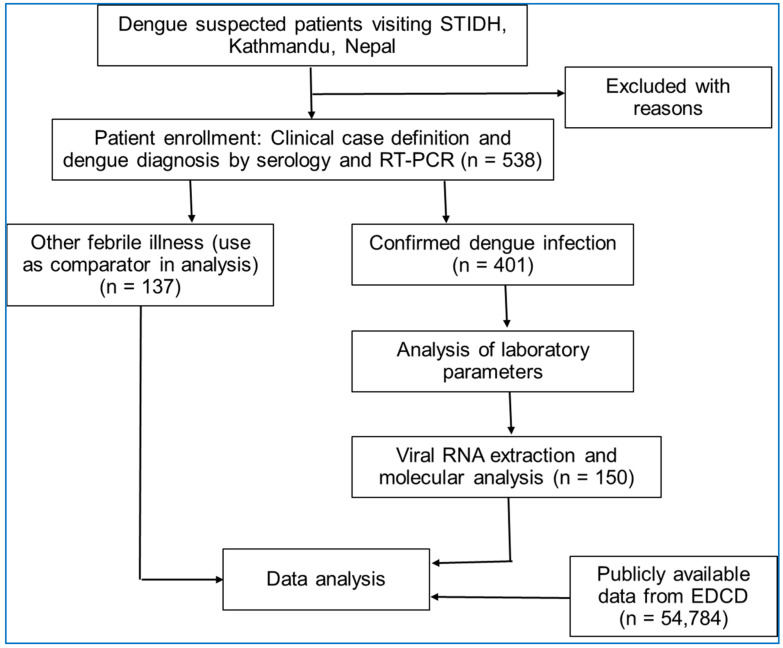
Flow diagram of the study showing patient enrollment, investigation and analysis approaches.

Dengue infection was diagnosed by detecting non-structural protein (NS1) antigen, IgM/IgG antibody (SD Bioline Dengue NS1 Duo Dengue NS1 Ag + Ab Combo, Standard Diagnostics, Suwon, Korea) and IgM enzyme-linked immunosorbent assay (ELISA; NovaLisa, NovaTec Immundiagnostica GmbH, Dietzenbach, Germany) as per the kit manufacturer’s instructions. The dengue case definition of the WHO was employed [3]. Dengue patients were followed up and classified as those requiring admission and those who recovered as outpatients. Additional clinical data and laboratory parameters were obtained from fever clinic logs and laboratory registers. Of the total 538 samples collected, 150 samples were randomly selected and subjected to molecular analysis (Figure 2). Patients below the age of 16 years were considered children in the analysis.

### 2.4. Viral RNA Extraction

Viral RNA was extracted using the QIAamp Viral RNA kit (QIAGEN, Hilden, Germany) directly from 140 μL of patient’s serum sample as per the manufacturer’s instructions. Viral RNA quantification and quality assessment were performed using NanoDrop (Thermo Fisher Scientific, Inc., Waltham, MA, USA).

### 2.5. One-Step Reverse Transcription Polymerase Chain Reaction (RT-PCR)

The presence of DENV RNA was detected by RT-PCR using the PrimeScript™ One Step RT-PCR Kit Ver. 2 (Takara Bio Inc., Shiga, Japan) following the manufacturer’s protocol. Briefly, the RT-PCR amplification was carried out in a final volume of 25 μL with 5 μL of RNA. The RT-PCR mixture consisted of 1 μL of enzyme mix, 13 μL of 2× buffer, 4 μL of nuclease-free water, and 1 μL of 100 pmol forward and reverse primers, with separate primer sets (Supplementary Table S1) for the detection of DENV and the identification of specific DENV serotypes [27,28,29]. The RT-PCR program consisted of the following steps: 50 °C for 30 min; 94 °C for 2 min; 30 cycles at 94 °C for 30 s, 54 °C for 30 s, 72 °C for 1 min; and 72 °C for 7 min; and carried out using the SimpliAmp Thermal Cycler (Applied Biosystems, Foster City, CA, USA). DNase/RNase-free water (Sigma, New York, NY, USA) served as a negative control, while a mixture of all four DENV serotypes (DENV-1 (99St12Astrain), DENV-2 (00St22A), DENV-3 (SLMC 50 strain), and DENV-4 (SLMC 318 strain)) with a known viral concentrations (10^6^ FFU/mL of DENV-1, -3, and -4 and 10^7^ pfu/mL of DENV-2) was used as a positive control [30]. Amplified products were detected by agarose gel electrophoresis and visualized in a UV chamber to confirm the presence of DENV or determine the DENV serotype.

### 2.6. Quantification of DENV Genome Levels

A volume of 5 µL of total RNA extracted from serum was used for quantitative real-time RT-PCR (qRT-PCR), and amplification of the envelope gene was performed as described previously [27,31]. Briefly, the envelope gene was amplified by using a total of 20 µL of reaction mixture consisting of 5 μL of Taqman master mix, 9 µL of nuclease-free water, 0.3 µL of 100 pmol forward and reverse primers (DENV serotype-specific), and 0.4 µL of probe (Supplementary Table S1) of TaqMan Fast Virus 1-Step Master Mix (Life Technologies, Carlsbad, CA, USA). The qRT-PCR program consisted of the following steps: 50 °C for 5 min, 95 °C for 20 s, and 40 cycles at 95 °C for 3 s and 60 °C for 30 s using the Quant Studio 5 real-time PCR system (Applied Biosystems, Foster City, CA, USA). The viral genome levels were expressed as log10 genome copies/mL.

### 2.7. Data Collection, Analysis and Statistics

Primary data were obtained from the patients enrolled at STIDH through questionnaires (demographic information), clinical records (fever clinic), and laboratory analysis (laboratory parameters and molecular information). Some data were also obtained from the public datasets made available by the EDCD, Ministry of Health, Nepal, to evaluate the national scenario. For EDCDs public datasets, dengue was diagnosed using rapid NS1/IgM/IgG assays. Data were entered into Microsoft Excel sheets, and, after proper cleaning and verification, they were exported to statistical analysis software (SPSS for Windows, version 22.0 (IBM Corp., Armonk, NY, USA) and GraphPad Prism 9 (Boston, MA, USA)). Continuous variables were presented as median (25–75% inter-quartile range (IQR)) and categorical variables as absolute number (n) and percentage (%). The comparison of continuous variables was performed using the Mann–Whitney U test and Kruskal–Wallis test between two and among three groups, respectively, whereas the chi-square test (or Fisher’s exact test when less than 5 in any cell) as appropriate was used to compare the categorical variables. Spearman’s correlation was used to analyze the correlation coefficient (rho). An alpha error of 0.05 was used to set statistical significance in all tests. 

## 3. Results

### 3.1. General Profiles of the Study Participants

We enrolled 538 febrile patients (age range 2–91 years, median (IQR) age was 30 years [22,23,24,25,26,27,28,29,30,31,32,33,34,35,36,37,38,39,40,41,42]) who were suspected of having dengue. Of these, 401 (74.5%) were diagnosed as laboratory-confirmed dengue cases, while the remaining patients had other febrile illnesses (OFI) of unknown origin(s). Dengue and non-dengue patients did not differ in their gender (*p* = 0.612) but they differed in age group (*p* = 0.005). As expected, dengue cases and OFI patients differed significantly (*p* < 0.05) in clinical features such as arthralgia, diarrhea, vomiting, retro-orbital pain, anorexia, white blood cells (WBC), neutrophils, platelets, mean corpuscular hemoglobin (MCH), mean corpuscular hemoglobin concentration (MCHC), serum urea, and alkaline phosphatase (ALP), and admission requirement, (Supplementary Tables S2 and S3). The majority of the dengue patients were adults (93.8%), with 6.2% being children. Most of the dengue patients who visited STIDH were from Kathmandu valley (55.6%, 223/401), while a notable proportion of patients were from the surrounding four districts and, to a lesser extent, referred cases from the other twelve distant districts, mostly from the Terai region (Figure 1).

### 3.2. Demographic, Clinical, and Laboratory Characteristics of Dengue Patients

Of the 401 dengue-confirmed patients (aged 29 years [22,23,24,25,26,27,28,29,30,31,32,33,34,35,36,37,38,39,40,41]), 129 patients (32.2%) required hospital admission. A higher proportion of adult patients (33%) required admission compared to children (12%; *p* = 0.027) and the median age for the inpatients and outpatients was 28 (22–43) and 30 (22–40) years, respectively. Several clinical and laboratory parameters were different between dengue patients who required hospital admission (inpatients) and non-admitted dengue patients who were managed as outpatients at the time of their first hospital visit. These significantly different parameters included myalgia, rash, diarrhea, retro-orbital pain, bleeding, and abdominal pain (*p* < 0.05), while the other parameters were not different (Table 1).

Similarly, patients requiring admission had significantly different hematological and biochemical parameters compared to the outpatient group at the time of their first hospital visit. Patients requiring admission had significant lymphocytosis, thrombocytopenia, and elevated liver enzymes (alanine aminotransferase [SGPT] and aspartate aminotransferase [SGOT]; *p* < 0.05, Table 2).

### 3.3. Spatiotemporal Distribution of Dengue Cases in Nepal, 2022

**Figure 3:**
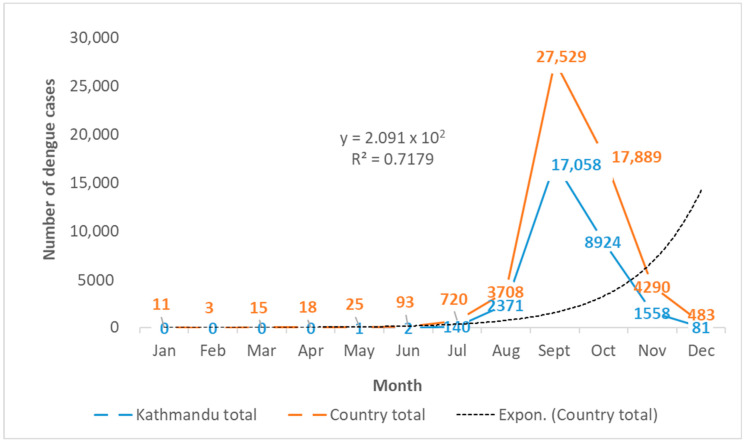
Temporal trend of dengue cases during the 2022 epidemic in Kathmandu (*n* = 30,135) and Nepal (*n* = 54,784). The dotted line is the trend line based on an exponential model of the country’s total dengue cases (*n* = 54,784). Data source: EDCD, Nepal [22].

During the 2022 dengue outbreak in Nepal, sporadic dengue cases started to appear in Nepal as early as January in the Terai region and in May in Kathmandu valley, based on publicly available EDCD data [22]. A gradual increase in cases led to a rapid surge that followed an exponential pattern from June in the country and July in Kathmandu, coinciding with the arrival of a monsoon (Figure 3). The epidemic peaked in September, with 50.3% (27,529/54,784) of the national total cases and 56.6% (17,058/30,135) of the total cases in Kathmandu occurring in a single month. Subsequently, the epidemic showed a declining trend in October, yet 17,889 cases throughout the country and 8924 cases in Kathmandu were reported in this month, which declined to a national total of 483 cases in December. This was in line with the delayed withdrawal of the monsoon and was a sign of the flattening of the epidemic. The Kathmandu valley (Kathmandu, 14,376; Bhaktapur, 9614; and Lalitpur, 6145) alone reported 55.0% of the total cases reported throughout the country. Cases were reported to the EDCD from all 77 districts of Nepal. A total of 88 patients died due to dengue out of 54,784 reported patients, and the case fatality rate (CFR) was 0.16% in 2022.

### 3.4. Multiple DENV Serotypes Circulated during the 2022 Epidemic in Nepal

**Figure 4:**
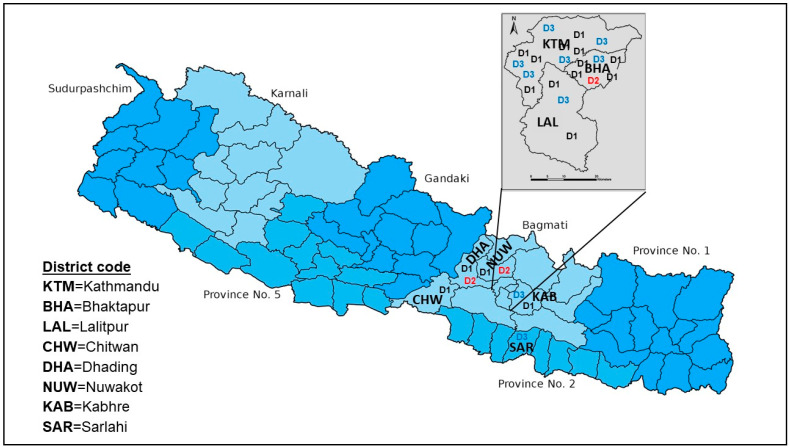
Spatial visual representation of the circulation of multiple DENV serotypes circulation in Kathmandu and surrounding districts in the 2022 outbreak. Regarding the molecular analysis of representative samples (*n* = 150), 29 strongly PCR-positive samples also had a positive serotype result. Kathmandu valley reported three DENV serotypes (DENV-1, DENV-2, and DENV-3), with the majority of patients being infected with DENV-1 and DENV-3.

Although this study focused on Kathmandu, the capital city of Nepal, we had the opportunity to analyze samples from patients referred to STIDH from other parts of the country. During this epidemic, we could confirm the circulation of three serotypes of DENV (DENV-1, -2, and -3), but not DENV-4 (Figure 4). Interestingly, all three reported serotypes circulated even in Kathmandu alone. Among the DENV PCR-positive patients (*n* = 29), the majority were infected with DENV-1 (57.1%) and DENV-3 (32.1%). Bhaktapur, the smallest district by land size, also reported all three serotypes. Other adjoining districts of Kathmandu also reported more than one serotype (Dhading and Nuwakot reported DENV-1 and DENV-2, while DENV-1 and DENV-3 were confirmed in Kabhre). Two lowland Terai districts, Chitwan and Sarlahi, had DENV-1 and DENV-3, respectively. One patient from Kathmandu valley (Bhaktapur district) had coinfection of DENV-1 and DENV-2.

### 3.5. DENV Viral Load during the 2022 Epidemic in Nepal

**Figure 5:**
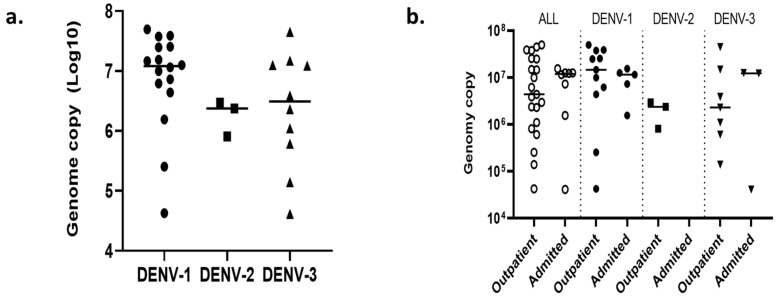
DENV viral load among different infecting serotypes and patient categories (*n* = 29). (**a**) Dot-plot of log genome copy number in different DENV serotypes and (**b**) dot-plot of log genome copy number among outpatients and admitted patients (inpatients). The Mann–Whitney U test and Kruskal–Wallis test were used to compare two groups (outpatients and inpatients) and three groups (DENV-1, -2, and -3), respectively.

Using the qRT-PCR approach, we next quantified the viral load in the serum of the selected dengue patients (*n* = 33) (Figure 5). Although the genome copy number among DENV serotypes was not significantly different, DENV-1 (median (IQR): 12,044,964.0 (4819,464.5–25,246,314.0)) had the highest average copy number (per mL) compared to DENV-2 (2373,748.0 (812,412.5–2665,839.6)) and DENV-3 (3091,965.3 (608,651.7–13,147,328.8); *p* = 0.139). No statistically significant difference was observed in the DENV genome copy numbers between outpatients and inpatients. However, almost all (except one patient) among the inpatient group had a high viral load, compared to the nearly equal distribution of low/high viral load in outpatients. There was a significant correlation between the Ct values and the DENV genome copy numbers for all three serotypes identified (r = 0.843, *p* < 0.001; Supplementary Table S4, Supplementary Figure S1). 

## 4. Discussion

Here, we reported the clinical characteristics and serotype distribution of the largest dengue epidemic in Nepal and identified DENV-1 and DENV-3 as the major serotypes (89.8%) responsible for this massive outbreak in 2022, while DENV-2 remained a minor serotype. Our findings based on the capital city Kathmandu and selected districts outside Kathmandu confirmed that the nationwide dengue epidemic was due to these two serotypes, although we also identified DENV-2 in some dengue patients. This is the first time that the co-circulation of at least three DENV serotypes (DENV-1 through -3) has been confirmed in the capital city Kathmandu, located at an altitude of 1300 m. Despite the periodic occurrence of major outbreaks in Nepal in 2006, 2010, 2013, 2018, and 2019, there are very limited data on the DENV serotypes circulating in the country (dominant serotypes: DENV-1/2, DENV-2, DENV-1, DENV-2, and DENV-2/3, respectively in 2010, 2013, 2016, 2017, and 2019) [15,16,17,32]. The available evidence suggests the circulation of multiple serotypes and serotype switching events between periodic dengue outbreaks in Nepal. All four serotypes were reported in 2006 in the country; however, the cases were sporadic in nature, with unclear travel history of a few patients, and multiple serotypes were not reported in a single location then [24]. It was during the 2010 major outbreak that all serotypes were reported (DENV-1 and DENV-2 as major serotypes) even from a single tropical location in the lowland Terai district (Chitwan), including multiple serotypes (DENV-1 and DENV-2) in Kathmandu [15]. DENV-2 also continued to establish in the Terai region in 2013 [33]. DENV-1 and DENV-3 in Terai and DENV-2 in a hill district (Dhading) near Kathmandu were reported in 2017 [32]. In the 2019 nation-wide dengue epidemic with over 14,000 cases [16], DENV-2 was the major serotype, while DENV-3 was a minor one [12]. Most of these studies had a common sampling limitation in relation to the sample size and geographic location restrictions. With these limitations, it is difficult to estimate the precise effects of serotype switching dynamics and their impact on population immunity and potential outbreak events. Nevertheless, the data certainly give an idea of how serotype-specific naïve populations existing in the country pose a risk of future outbreaks. For instance, the Kathmandu patterns of DENV-1 (only a few cases in 2010) [15] and DENV-3 (never reported before) may have favored the 2022 epidemic with the reemergence of DENV-1 in Nepal after several years. One serotype confers lifetime immunity to that infecting serotype [1,34,35], and this phenomenon implies to other parts/populations of the country apart from Kathmandu. This situation calls for urgent action for the molecular surveillance of DENV throughout the country every year. Integrated disease surveillance (IDS) for arboviruses, as proposed earlier, is one appropriate approach [14,15].

This was the most devastating dengue epidemic ever reported in Nepal, with 54,784 cases and 88 deaths in 2022. Kathmandu was the worst-hit valley due to this epidemic, reporting 55.3% of the national total cases. Fatality due to dengue was rare in Nepal during the previous outbreaks, with the highest number of deaths (six) reported in 2019 (CFR, 0.04%) followed by four deaths in 2010 (CFR, 0.44%) [15,16,25,33]. Although the reported CFR (0.16%) was still low in 2022, the size of the dengue epidemic was massive, resulting in a huge number of casualties, and the total number of deaths in 2022 alone is far higher than the cumulative dengue-related deaths reported since the introduction of DENV in Nepal. The proportion of hospitalized patients (32.2%) indicates an unprecedented burden to hospitals, which likely impacted the clinical management or access to hospitalization during the epidemic. This catastrophic situation was probably the result of population biology/immune response consequences, vector adaptation, and climate change. More importantly, evidence of the circulation of multiple DENV serotypes in the major epidemic areas in the past might be one reason for severe dengue and fatalities, due to short-term cross-protective immunity, infection time interval, and antibody-dependent enhancement (ADE) in the secondary infections [36,37,38]. 

The majority (93.8%) of the dengue patients were adults during the 2022 epidemic. The age-wise incidence of dengue in Nepal (6.2% being children in the current epidemic) largely differs from that of other endemics in Asian countries, where children are primarily affected [39]. This is probably due to the relatively short history of dengue establishment in the country, with the first major outbreak reported only in 2010 [15,16]. The risk of clinical dengue is dependent on the age after primary infection [40], relating it to mostly adult cases, like in Nepal. If we look at the trend of the child dengue cases in Nepal, a gradual increase in the number of child dengue cases over the past several years is observed, although the majority of dengue patients were adults [12,15,17,41]. This can also be explained by the gradually growing force of the infection, due to the greater availability of naïve populations in the areas with recent dengue introduction [42]. Therefore, the shape of future dengue epidemics in Nepal may skew toward a younger population/children when the force of infection becomes sufficiently high as the country becomes hyper-endemic, posing an even greater risk of severity and deaths. With such a massive epidemic with cases reported from all 77 districts of the country, Nepal needs to evaluate the age-related incidence and force of infection by employing well-designed cohort studies to support dengue control and vaccine strategies.

Interestingly, a higher proportion of dengue patients during the 2022 epidemic had diarrhea, vomiting, myalgia, anorexia, and retro-orbital pain compared to the previous outbreaks in Nepal, while elevated liver enzymes, lymphocytosis, and thrombocytopenia were significant in inpatients as in the previous outbreaks [12,15,41]. A few dengue patients also had unusual central nervous system (CNS) involvement, as has been reported elsewhere [15,27,43]. Higher frequencies of warning signs and some severe dengue signs can be linked with relatively higher hospitalization and deaths in 2022 [3]. The DENV viral load dynamics in 2022 patients showed that all patients (except one) among the inpatient group had a high viral load compared to the nearly uniform distribution of genome copy numbers in outpatients. This indicates that higher viremia propelled the severity in dengue [44]. Infection with DENV-1 produced higher viremia levels compared to DENV-2 and -3 in the 2022 outbreak in Nepal, which is likely associated with the reemergence of DENV-1 after several years and its ADE, leading to enhanced infectivity, severity, and deaths [36,37,38]. 

Although we do not have direct evidence, a dengue epidemic of this magnitude and its rapid expansion to higher altitudes might be linked with climate change and vector abundance, despite the fact that dengue outbreaks of small-to-moderate scale occur every 2–3 years in Nepal [45,46,47,48]. Meteorologists forecasted above-normal monsoon rains in 2022, and the number of dengue cases corroborated this peak monsoon. This is another dimension of dengue that requires further investigation for epidemic preparedness through informed policy-making in Nepal. The dengue epidemic further alerts the public health authorities to work in vector control, environmental and clinical management, and vaccination strategies when available.

## 5. Conclusions

We confirmed the circulation of three DENV serotypes during the massive 2022 outbreak in Nepal, with a dominance of DENV-1 and -3. This indicates serotype displacement events; in particular, DENV-1 reemerged after several years in the country and exhibited high viral loads. This epidemic had a higher frequency of hospital admissions, more severe dengue, and more deaths. Kathmandu, located at an altitude of 1300 m above sea level, remained the most affected area. The magnitude and geographical scope of expansion of dengue in Nepal are real challenges to control program. Therefore, the precise mapping of the DENV infection through IDS (along with other arboviruses), the dynamics of population-level immunity, and virus evolution should be urgent plans of action for evidence-based policy-making for dengue control and prevention in the country. 

## Figures and Tables

**Table 1:** Demographic and clinical characteristics of dengue patients, Nepal, 2022.

Characteristics	Category	Outpatients	Inpatients	*p*-Value
**Age**	Child	22 (88.0)	3 (12.0)	**0.027**
	Adult	250 (66.5)	126 (33.5)	
**Gender**	Female	106 (64.2)	59 (35.8)	0.198
	Male	166 (70.3)	70 (29.7)	
**Travel history**	No	32 (24.1)	101 (75.9)	1.000
	Yes	1 (14.3)	6 (85.7)	
Fever *	Yes	272 (67.8)	129 (32.2)	-
**Myalgia**	No	26 (89.7)	3 (10.3)	**0.046**
	Yes	246 (66.1)	126 (33.9)	
**Rash**	No	238 (73.7)	85 (26.3)	**<0.001**
	Yes	34 (43.6)	44 (56.4)	
**Diarrhea**	No	247 (72.6)	93 (27.4)	**<0.001**
	Yes	25 (41.0)	36 (59.0)	
**Vomiting**	No	34 (64.2)	19 (35.8)	0.538
	Yes	238 (68.4)	110 (31.6)	
**Retro-orbital pain**	No	239 (71.8)	94 (28.2)	**<0.001**
	Yes	33 (49.3)	34 (50.7)	
**Any bleeding**	No	270 (71.3)	109 (28.7)	**<0.001**
	Yes	2 (9.1)	20 (90.9)	
**Anorexia**	No	7 (77.8)	2 (22.2)	0.724
	Yes	265 (67.6)	127 (32.4)	
**Sore throat**	No	258 (68.0)	126 (32.0)	0.288
	Yes	14 (82.4)	3 (17.6)	
**Abdominal pain**	No	269 (69.5)	118 (30.5)	**<0.001**
	Yes	3 (21.4)	11 (78.6)	
**Ascites**	No	33 (24.1)	104 (75.9)	1.000
	Yes	0 (0)	3 (100.0)	
**Hepatomegaly**	No	33 (24.3)	103 (75.7)	0.573
	Yes	0 (0)	4 (100.0)	
**Splenomegaly**	No	33 (23.7)	106 (76.3)	1.000
	Yes	0 (0)	1 (100.0)	

The chi-square test was used to analyze categorical variables. Figures in the parentheses indicate percentages unless stated otherwise. * All were febrile patients.

**Table 2:** Blood parameter profiles of dengue patients, Nepal, 2022.

Blood Parameters	Outpatient, Median (IQR)	Inpatient, Median (IQR)	*p*-Value
**Hemoglobin**	13.6 (12.3–14.8)	13.7 (12.5–14.7)	0.608
**Total count (WBC)**	3700 (2800–5300)	3300 (2300–5250)	0.166
**Neutrophils**	61.5 (50.0–71.0)	56.0 (40.5–70.5)	0.063
**Lymphocytes**	29.0 (20.0–39.8)	33.0 (21.0–45.5)	**0.024**
**Eosinophils**	0.5 (0–1.0)	1.0 (0–2.0)	0.608
**Monocytes**	8.0 (5.0–10.0)	9.0 (5.0–10.0)	0.530
**Platelets**	135.0 (96.3–187.8)	72.0 (46.0–120.0)	**<0.001**
**HCT**	41.0 (37.0–45.0)	40.0 (37.0–44.0)	0.348
**RBC**	4.8 (4.3–5.2)	4.7 (4.3–5.2)	0.223
**MCV**	86.0 (82.0–90.0)	85.0 (81.0–88.0)	0.203
**MCH**	29.0 (27.0–30.0)	29.0 (28.0–30.0)	**0.016**
**MCHC**	33.0 (32.0–34.0)	34.0 (33.0–35.0)	**<0.001**
**Serum urea**	24.0 (21.0–28.0)	19.0 (16.0–28.8)	0.202
**Creatinine**	1.0 (0.6–1.1)	0.8 (0.6–1.0)	0.493
**Sodium**	138.0 (135.0–140.5)	136.0 (132.8–137.0)	0.095
**Potassium**	4.0 (3.7–4.0)	3.9 (3.6–4.3)	0.837
**SGPT**	21.0 (17.5–27.5)	54.0 (27.0–121.0)	**0.004**
**SGOT**	24.0 (22.0–49.0)	84.0 (49.0–183.0)	**0.003**
**ALP**	90.5 (67.5–99.3)	74.0 (63.0–86.0)	0.345
**Bilirubin-total**	0.7 (0.6–0.7)	0.7 (0.7–0.9)	0.173
**Bilirubin-direct**	0.2 (0.1–0.2)	0.2 (0.1–0.2)	0.932

The Mann–Whitney U test was used to compare continuous variables between two groups. IQR, inter-quartile range; WBC, white blood cells; HCT, hematocrit; RBC, red blood cells; MCV, mean corpuscular volume; MCH, mean corpuscular hemoglobin; MCHC, mean corpuscular hemoglobin concentration; SGPT, alanine aminotransferase; SGOT, aspartate aminotransferase; and ALP, alkaline phosphatase.

## Data Availability

The datasets generated and/or analyzed during the current study are available in the manuscript and the Supplementary Materials.

## Supplementary Materials

The following supporting information can be downloaded at: https://www.mdpi.com/article/10.3390/v15020507/s1. Table S1. Dengue conventional and real time RT-PCR primers and probes; Table S2. Demographic and clinical parameters of dengue patients and OFI patients; Table S3. Laboratory parameters of dengue patients and OFI patients; Table S4a. Ct Values and genome copy numbers in different serotypes infection; Table S4b. Ct value and genome copy number among different serotypes; Table S4c. Correlation between Ct and genome copy number; Figure S1. Scatter-plot with Spearman’s correlation between log genome copy number and their respective Ct values.
