# Supplementary material for: Co-Circulation of Dengue Virus Serotypes 1, 2, and 3 during the 2022 Dengue Outbreak in Nepal: A Cross-Sectional Study
Source: Viruses. 2023 Feb 11;15(2):507. doi: 10.3390/v15020507 (PMC9958792; doi:10.3390/v15020507)

Supplementary Table S1. Dengue conventional and real time RT-PCR primers and probes

Assay	Identification	Sequences (5' - 3')	Size (base pair)
Conventional RT-PCR	**Dengue Consensus primer**		
	DC-1, Forward	TCAATATGCTGAAACGCGCGAGAAACCG	511
	DC-2, Reverse	TTGCACCAACAGTCAATGTCTTCAGGTTC	
	**Dengue serotype primer**		
	D1-Forward	GGACTGCGTATGGAGTTTTG	490
	D1-Reverse	ATGGGTTGTGGCCTAATCAT	
	D2-Forward	GTTCCTCTGCAAACACTCCA	230
	D2-Reverse	GTGTTATTTTGATTTCCTTG	
	D3-Forward	GTGCTTACACAGCCCTATTT	320
	D3-Reverse	TCCATTCTCCCAAGCGCCTG	
	D4-Forward	CCATTATGGCTGTGTTGTTT	399
	D4-Reverse	CTTCATCCTGCTTCACTTCT	
Dengue serotype specific Real time RT-PCR	D1-469 Forward	GAACATGGRACAAYTGCAACYAT	67
	D1-536 Reverse	CCGTAGTCDGTCAGCTGTATTTCA	
	MGB-493 probe	ACACCTCAAGCTCC	
	D2-493 Forward	ACACCACAGAGTTCCATCACAGA	68
	D2-568 Reverse	CATCTCATTGAAGTCNAGGCC	
	MGB-545 probe	CGATGGARTGCTCTC	
	D3-1 Forward	ATGAGATGYGTGGGAGTRGGAAAC	70
	D3-71 Reverse	CACCACDTCAACCCACGTAGCT	
	MGB-27 probe	AGATTTTGTGGAAGGYCT	
	D4-711 Forward	GGTGACRTTYAARGTHCCTCAT	75
	D4-786 Reverse	WGARTGCATRGCTCCYTCCTG	
	TAMPRA-734 probe	CCAAGAGACAGGATGTGACAGTGCTRGGATC	

Table S2. Demographic and clinical parameters of dengue patients and OFI patients

Characteristics	Category	Dengue patients	OFI patients	P-value
**Age**	Child	25 (56.8)	19 (43.2)	**0.005**
	Adult	376 (76.1)	118 (23.9)	
**Gender**	Female	165 (75.7)	53 (24.3)	0.612
	Male	236 (73.8)	84 (26.2)	
**Travel history**	No	133 (93.0)	10 (7.0)	1.000
	Yes	7 (100.0)	0 (0.0)	
**Fever**	Yes	401 (74.5)	137 (25.5)	-
**Myalgia**	No	29 (72.5)	11 (27.5)	0.759
	Yes	372 (74.7)	126 (25.3)	
**Rash**	No	323 (72.3)	124 (27.7)	0.07
	Yes	78 (85.7)	13 (14.3)	
**Diarrhea**	No	340 (71.9)	130 (28.1)	**<0.001**
	Yes	61 (93.8)	4 (6.2)	
**Vomiting**	No	53 (36.6)	92 (63.4)	**<0.001**

	Yes	348 (88.5)	45 (11.5)	
**Retro-orbital pain**	No	333 (71.8)	131 (28.2)	**<0.001**
	Yes	67 (91.8)	6 (8.2)	
**Any bleeding**	No	378 (73.5)	136 (26.5)	0.672
	Yes	23 (95.8)	1 (4.2)	
**Anorexia**	No	9 (36.0)	16 (64.0)	**<0.001**
	Yes	392 (76.4)	121 (23.6)	
**Sore-throat**	No	384 (75.1)	127 (24.9)	0.157
	Yes	17 (63.0)	10 (37.0)	
**Abdominal pain**	No	387 (74.9)	130 (25.1)	0.398
	Yes	14 (66.7)	7 (33.3)	
**Ascites**	No	137 (93.2)	10 (6.8)	1.000
	Yes	3 (100.0)	0 (0.0)	
**Hepatomegaly**	No	136 (93.2)	10 (6.8)	1.000
	Yes	4 (100.0)	0 (0.0)	
**Splenomegaly**	No	139 (93.9)	10 (6.1)	1.000

Yes	1 (100.0)	0 (0.0)	

Chi-square test was used to analyze categorical variables. Figures in the parentheses indicate percentage unless stated otherwise. P<0.05 was considered statistically significant.

Supplementary Table S3. Laboratory parameters of dengue patients and OFI patients

Blood parameters	Dengue patients, Median (IQR)	OFI patients, Median (IQR)	P-value
**Hemoglobin**	13.6 (12.3-14.8)	13.7 (12.4-14.7)	0.897
**Total count (WBC)**	3600 (2600-5300)	6100 (4650-8200)	**<0.001**
**Neutrophils**	61.0(48.0-71.0)	64.0 (51.0-75.0)	**0.033**
**Lymphocytes**	30.0 (20.0-30.0)	28.0 (15.5-39.0)	0.060
**Eosinophils**	1.0 (0-1.0)	1.0 (0-2.0)	0.063
**Monocytes**	8.0 (5.0-10.0)	8.0 (5.0-10.0)	0.057
**Platelets**	116.0 (72.5-172.0)	198.0 (145.0-257.0)	**<0.001**
**HCT**	41.0 (37.0-45.0)	41.0 (38.0-45.0)	0.307
**RBC**	4.7 (4.3-5.2)	4.8 (4.4-5.3)	0.089

**MCV**	86.0 (82.0-89.5)	86.0 (82.0-89.0)	0.866
**MCH**	29.0 (27.0-30.0)	28.0 (26.0-30.0)	**0.019**
**MCHC**	34.0 (32.0-35.0)	33.0 (31.0-35.0)	**0.029**
**Serum urea**	19.0 (17.0-28.0)	12.0 (6.3-13.5)	**0.030**
**Creatinine**	0.8 (0.6-1.0)	0.8 (0.4-0.9)	1.000
**Sodium**	136.0 (133.0-137.0)	138.0 (124.0-141.5)	0.607
**Potassium**	3.9 (3.6-4.3)	3.8 (3.7-23.4)	0.644
**SGPT**	45.5 (25.2-114.7)	30.5 (17.7-89.0)	0.331
**SGOT**	75.0 (42.2-173.2)	36.0 (26.0-105.0)	0.423
**ALP**	75.0 (63.0-91.0)	107.0 (106.0-124.0)	**0.010**
**Bilirubin-Total**	0.7 (0.7-0.9)	0.6 (0.6-0.8)	0.524
**Bilirubin-Direct**	0.2 (0.1-0.2)	0.1 (0.1-0.2)	0.645

Mann Whitney U test was used to compare two continuous variables between two groups. P<0.05 was considered statistically significant. IQR, inter-quartile range; WBC, white blood cells; HCT, hematocrit; RBC, red blood cells; MCV, mean corpuscular volume; MCH, mean corpuscular hemoglobin; MCHC, mean corpuscular hemoglobin concentration; SGPT, alanine aminotransferase; SGOT, aspartate aminotransferase; ALP, alanine phosphatase.

Supplementary Table S4a. Ct Values and genome copy numbers in different serotypes infection

Sample ID	District	DENV serotype	Ct value	Genome copies/ml
10726	Nuwakot	DENV-1	19.6	7,256,242
10937	Kabhre	DENV-3	23.237	3,884,472
11011	Bhaktapur	DENV-1	19.9	6,176,926
11539	Kathmandu	DENV-1	20.4	4,366,978
15312	Kathmandu	DENV-1	16.7	49,587,604
15719	Kabhre	DENV-1	17.2	37,483,436
16840	Sarlahi	DENV-3	28.220	140,134
16880	Kathmandu	DENV-1	17.1	38,776,880
17002	Kathmandu	DENV-3	21.513	12,266,968
17084	Kathmandu	DENV-3	24.023	2,299,458
18085	Kathmandu	DENV-1	18.6	14,650,465
18367	Lalitpur	DENV-1	17.7	25,353,902

18472	Kathmandu	DENV-3	25.129	1,100,126
31338	Kathmandu	DENV-1	19.2	9,967,617
31391	Kathmandu	DENV-3	26.017	608,652
31400a	Bhaktapur	DENV-1	27.4	42,533
31400b	Bhaktapur	DENV-2	19.2	2,373,748
31485	Dhading	DENV-1	24.7	254,316
31509	Lalitpur	DENV-1	18.8	12,554,234
31552	Kathmandu	DENV-1	17.8	24,923,550
5093	Kathmandu	DENV-3	21.474	12,586,220
5265	Lalitpur	DENV-3	21.228	14,830,655
7366	Nuwakot	DENV-2	19.0	2,957,931
8771	Dhading	DENV-2	20.4	812,413
9196	Bhaktapur	DENV-3	19.573	44,697,648
9208	Bhaktapur	DENV-1	22.0	1,553,338
9338	Kathmandu	DENV-3	30.057	41,150
9391	Bhaktapur	DENV-1	18.9	11,535,694

9685	Chitwan	DENV-1	18.5	15,424,199

Supplementary Table S4b. Ct value and genome copy number among different serotypes

	DENV-1	DENV-2	DENV-3	P-value
**Ct, Median (IQR)**	18.9 (17.8-20.3)	19.2 (18.9-19.8)	23.6 (21.4-26.6)	**0.003**
**Genome copy number, Median (IQR)**	12044964.0 (4819464.5-25246314.0)	2373748.0 (812412.5-2665839.6)	3091965.3 (608651.7-13147328.8)	0.139

Supplementary Table S4c. Correlation between Ct and genome copy number

	Spearman's correlation coefficient (rho)		P-value
	**Ct value**	**Genome copy number**	
**Ct value**	1.000	-.843**	**<0.001**
**Genome copy number**	-.843**	1.000	

**. Correlation is significant at the 0.01 level (2-tailed).

Supplementary Figure S1. **Scatter-plot with Spearman's correlation between log genome copy number and their respective Ct values**

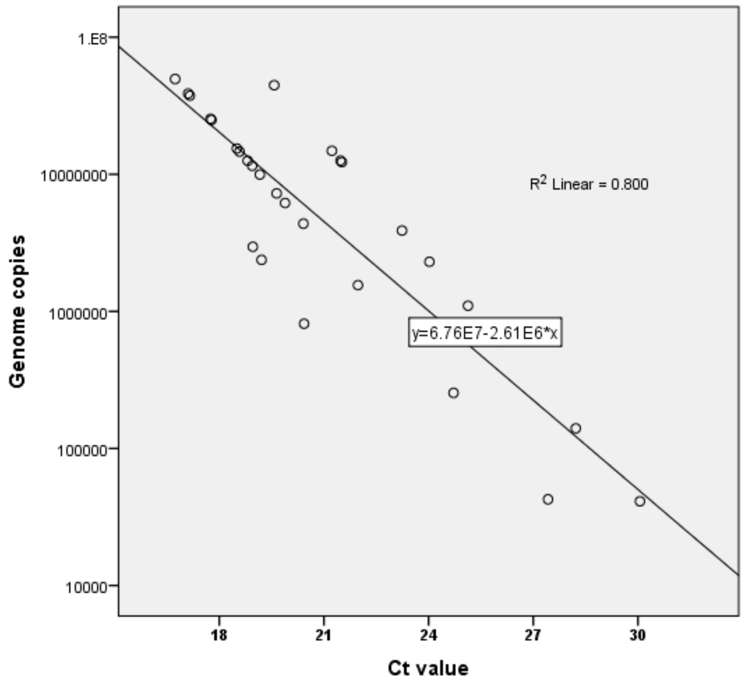

Supplement: Supplementary file 1 [file viruses-15-00507-s001.zip › viruses-2138717-supplementary.pdf]
